# How does health visiting in the first year of life vary by family characteristics? A longitudinal analysis of administrative data

**DOI:** 10.1093/pubmed/fdae259

**Published:** 2024-09-16

**Authors:** C Bunting, A Clery, L McGrath-Lone, M Liu, S Kendall, H Bedford, F Cavallaro, E C Saloniki, K Harron, J Woodman

**Affiliations:** UCL Great Ormond Street Institute of Child Health, University College London, London WC1N 1EH, UK; UCL Great Ormond Street Institute of Child Health, University College London, London WC1N 1EH, UK; Social Research Institute, University College London, London WC1H 0AA, UK; UCL Great Ormond Street Institute of Child Health, University College London, London WC1N 1EH, UK; Centre for Health Services Studies, University of Kent, Canterbury CT2 7NF, UK; UCL Great Ormond Street Institute of Child Health, University College London, London WC1N 1EH, UK; Data Analytics Team, The Health Foundation, London EC4Y 8AP, UK; Department of Primary Care and Population Health, University College London, London NW3 2PF, UK; Research Partnership Team, National Institute for Health and Care Research Applied Research Collaboration North Thames, London W1T 7HA, UK; UCL Great Ormond Street Institute of Child Health, University College London, London WC1N 1EH, UK; Social Research Institute, University College London, London WC1H 0AA, UK

**Keywords:** children, health services, public health

## Abstract

**Background:**

The health visiting service in UK promotes the health and wellbeing of families with young children and comprises a universal offer (three mandated contacts between birth and 12 months) and additional contacts based on need. We aimed to understand how the level of health visiting support received varies by family characteristics.

**Methods:**

Using the Community Services Data Set linked to Hospital Episode Statistics, we identified 52 555 children in 10 local authorities with complete health visiting data for 12 months between April 2016 and March 2020. We analysed variation in health visiting contacts by deprivation, child ethnicity, maternal age, adversity and previous live births.

**Results:**

41 340/52 555 children (79%) received the universal service; 63% received ≥1 additional contact and 25% received ≥3 additional contacts. The likelihood of receiving ≥3 additional contacts was greatest for children whose mothers had a history of hospital admissions relating to mental health, violence, self-harm or substance misuse (adjusted relative risk = 1.55, 95% confidence interval 1.26–1.92).

**Conclusions:**

Most families received health visiting support in addition to the universal service. Policymakers and commissioners should consider how health visiting services can be expanded or targeted more effectively to ensure all families receive the support they need.

## Introduction

Health visiting is a well-established service that aims to maximize the health and wellbeing of children through supporting families and joint working with other services.^1^ In UK, health visitors lead the Healthy Child Programme for children aged 0–5 years which includes health screening, immunization, health and development reviews and parenting advice and support.^2–4^

Health visiting in UK is designed according to a model of proportionate universalism: every family receives some support with those who need more, receiving more.^5^ The universal element of the Healthy Child Programme comprises five mandated health visiting contacts for every child and family: at 28 weeks pregnancy, 10–14 days after birth, 6–8 weeks, 9–12 months and 2–2½ years. Many families’ needs will be met by assessment, guidance, and signposting within these mandated contacts. However, other families will need more targeted support which may comprise additional health visiting contacts or referrals to other services.

Since 2015, local authorities (LAs) in UK have been responsible for the Healthy Child Programme, including commissioning of health visiting. Aggregate data on the number of mandated health visiting contacts delivered by LAs is published quarterly as the Health Visitor Service Delivery Metrics.^6^ There are no equivalent metrics for the number of additional contacts delivered by LAs.

Recent studies have used the Community Services Dataset (CSDS), an individual-level dataset with information about publicly funded community services,^7^ to investigate how support is provided by health visiting teams in addition to the mandated service. Our analysis of 1.8 million health visiting contacts for children aged under five across 57 LAs between 2018 and 2020 found 80% of LAs delivered more additional than mandated contacts: an average 1.6 additional contacts were delivered for each mandated contact, ranging from 0.1 to 8.5 across all LAs.^8^ Other research has found that children living in deprived areas receive fewer mandated but more additional contacts.^7^^,^^9^^,^^10^

These cross-sectional analyses show how health visiting contacts were provided to populations at different time points. To plan services and assess workforce requirements, policymakers and commissioners also need to understand how families interact with health visiting teams over time and which families, at which life stages, need most support. This study used CSDS linked to hospital records to describe how health visiting contacts were delivered during the first year of life in 10 LAs in UK between 2016 and 2020 and how the amount of support provided varies by family characteristics.

## Methods

### Data sources

The CSDS is a national dataset capturing data on health visiting contacts received by children in UK, including the date and duration of contact, and demographic information such as child sex and ethnicity.^7^ Hospital Episode Statistics (HES) provide data on NHS-funded hospital admissions, including type (e.g. emergency, elective) and date of admission and clinical information based on International Classification of Diseases 10th Revision (ICD-10) diagnostic codes.^11^ When a child is born in an NHS hospital, a birth record is created within HES. NHS England assigns the same unique identifier to children in CSDS and HES, allowing us to link these two data sources together.^12^ A mother-baby link connects children to their mothers in HES, allowing us to access information on the medical history of mothers.^13^ We also accessed delivery records, which are assigned to mothers in HES and include information on the birth of each child such as birthweight and gestational age.

### Study population

The study population was children born in UK between April 2016 and March 2019, based on month and year of birth and first LA of residence, as recorded in CSDS. From CSDS, we extracted all health visiting contacts recorded between 2016/17 (the earliest year for which CSDS data are available) and 2019/20 (the last financial year before the COVID-19 pandemic, during which health visiting services were disrupted).^14^ We followed up each child and their health visiting contacts that took place in the first 12 months of life.

### Identifying type of health visiting contact

We used CSDS activity type codes 8–10 to flag mandated contacts in the first 12 months (new birth visit, 6–8-week review and 1-year review). Where activity type was missing (30% of records), we defined a contact as mandated if it took place within a plausible time window for each mandated contact. All other health visiting contacts were categorized as additional contacts.^15^

### Identifying a sample of LAs with good quality CSDS data

CSDS is compiled by aggregating health visiting delivery data provided by LAs. All 149 upper tier LAs in UK are included in CSDS (Isles of Scilly combined with Cornwall; City of London combined with Hackney), but the completeness of data submitted to CSDS varies by LA and over time. We restricted our sample to 10 LAs that had complete data in CSDS relative to external reference data for at least five consecutive financial quarters between April 2016 and March 2020.^15^ We identified children born in the relevant time window in each of the 10 LAs to ensure complete health visiting data for 12 months. We excluded children who moved LA before age 1, as they may have moved to an LA with incomplete health visiting data.

Our final cohort contained all health visiting contacts by age one for 52 555 children born in NHS hospitals and living within 10 LAs at different time points between April 2016 and March 2020, who were linked to their mother’s delivery record in HES (Supplementary Fig. 1, Supplementary Table 1).

### Family characteristics

We analysed variation in health visiting contacts by key family characteristics: deprivation, child ethnicity, maternal age, maternal adversity and whether the child was the mother’s first live birth. Deprivation was measured by the decile of the Index of Multiple Deprivation (IMD), derived from the residential postcode of the child, as recorded in CSDS. Child ethnicity is categorized in CSDS as Census categories: White, Mixed, Asian, Black or Other. Maternal age was taken from the mother’s delivery record in HES and categorized into < 20, 20–24, 25–29, 30–34, 35–39 and 40+ years. We used the number of previous live births on delivery records to identify first-time mothers. Maternal adversity was derived from ICD-10 diagnosis codes recorded in maternal hospital admission records in the 3 years prior to delivery. We considered codes relating to mental health conditions, violence, self-harm or substance misuse as indicators of adversity (Supplementary Table 2), based on previously published code lists.^16^

### Statistical analysis

We calculated the percentage of children receiving all three mandated contacts, at least one additional contact and three or more additional contacts between birth and 12 months. Where data were available, we also described duration of contacts. We explored how the number of contacts varied according to deprivation, child ethnicity, maternal age, maternal adversity, and whether the child was the mother’s first live birth. To determine which characteristics were associated with receiving different numbers of contacts, we used generalized linear models to estimate relative risks, adjusted for all covariates. Multi-level models were used to account for clustering within LAs, and robust standard errors were used to account for clustering of children within mothers. All results were rounded to the nearest five to align with NHS England guidance on statistical disclosure control.

## Results

### Characteristics of our LA cohort

The 10 LAs included in our sample differed from the 149 LAs across UK (Supplementary Table 3). Only one of the 10 LAs was in the most deprived quintile of IMD while three LAs were in the least deprived quintile. The study LAs also had a higher proportion of population who were White (93.0% compared to 85.3% nationally) and a lower proportion of all other ethnic groups (e.g. 3.7% Asian compared to 7.2% nationally). The 10 LAs did not include representation from London or the North East of UK.

### Amount of health visiting support received

**Fig. 1:**
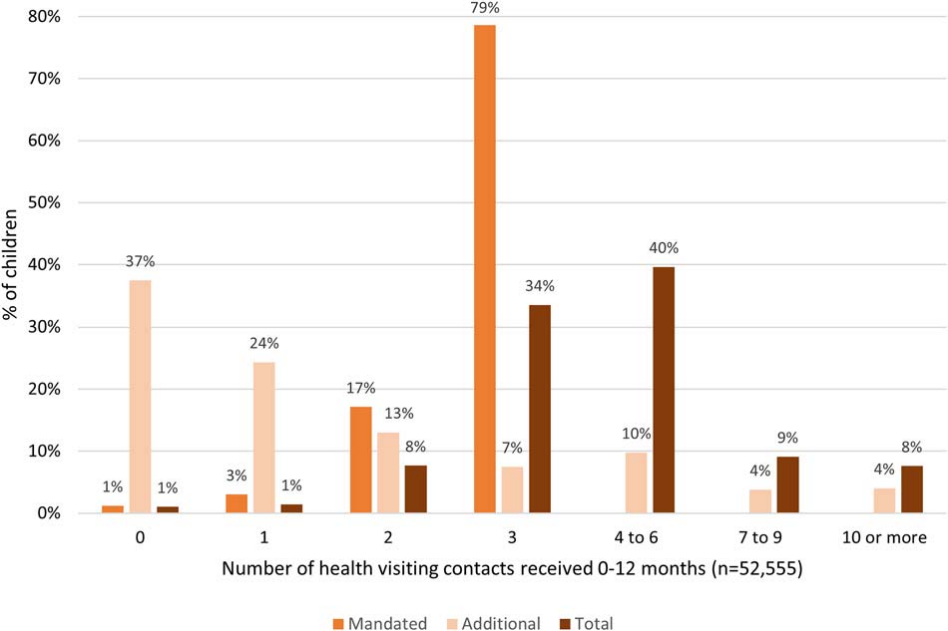
Percentage of children receiving mandated and additional health visiting contacts between birth and age 1 for children in 10 English local authorities between 2016/17 and 2019/20

Almost all children in our sample (99.0%) saw the health visiting team at least once within the first year of life (Fig. 1). Mandated postnatal contacts were consistently delivered, with most (78.7%) children receiving all three and 98.8% receiving at least one. The number of total contacts varied across children; the median number per child was four (interquartile range (IQR) 3–5). Most children (62.5%) received at least one additional contact in their first year of life, a quarter (25.1%) had ≥3 and 4% had ≥10.

Amongst 50 985 children who saw the health visiting team at least once and had complete duration data for all contacts, the median amount of time spent with the health visiting team during the first year of life was 2 h 35 min (IQR 120–205 min). The median duration of total contacts for children who had all mandated contacts was 2 h 45 min (IQR 135–215 min) compared with 3 h (IQR 140–252 min) in those with at least one additional contact and 4 h 30 min (IQR 195–410 min) in those with at least three additional contacts. Children who received 10 or more additional contacts had a median cumulative duration of 11 h 5 min contact from birth to 12 months (IQR 375–1037 min).

### Variation in health visiting support by family characteristics

Mandated contacts were delivered consistently to children across all levels of deprivation (Table 1). The likelihood of receiving additional contacts increased with level of deprivation: 42.4% of children in the most deprived areas received three or more additional contacts in the first 12 months, compared to 17.2% in the least deprived.

**Table 1:** Characteristics of children receiving health visiting contacts between birth and age 1 for children in 10 English local authorities between 2016/17 and 2019/20

	Total^a^	Received all 3 mandated contacts		Received ≥ 1 additional contact		Received ≥ 3 additional contacts	
	N	N	%	N	%	N	%
**Total**	52 555	41 340	78.7	32 860	62.5	13 195	25.1
**Index of Multiple Deprivation decile**							
Most deprived	2290	1860	81.2	1790	78.2	970	42.4
2	3620	2870	79.3	2615	72.2	1205	33.3
3	3740	2900	77.5	2625	70.2	1205	32.2
4	4120	3295	80.0	2725	66.1	1340	32.5
5	5150	4000	77.5	3370	65.5	1450	28.2
6	5180	4005	77.4	3135	60.6	1160	22.4
7	5730	4505	78.6	3505	61.1	1385	24.1
8	6000	4720	78.7	3660	61.0	1365	22.8
9	7560	5985	79.2	4410	58.3	1535	20.3
Least deprived	9165	7210	78.6	5025	54.8	1580	17.2
**Child ethnicity**							
White	41 065	32 755	79.8	25 570	62.3	10 360	25.2
Mixed	4080	3225	79.0	2860	70.1	1150	28.2
Asian	1710	1360	79.5	1015	59.4	325	19.0
Black	645	495	76.7	400	62.0	185	28.7
Other	545	400	73.4	315	57.8	115	21.1
**Maternal age (years)**							
<20	1300	945	72.7	1005	77.3	635	48.8
20–24	6745	5125	76.0	4480	66.4	1990	29.5
25–29	14 880	11 705	78.7	9340	62.8	3675	24.7
30–34	17 845	14 245	79.8	10 855	60.8	4140	23.2
35–39	9690	7675	79.2	5805	59.9	2185	22.5
40+	2095	1645	78.5	1370	65.4	575	27.4
**First-time mother**							
No	28 125	21 760	77.4	16 895	60.1	6355	22.6
Yes	24 430	19 575	80.1	15 965	65.3	6840	28.0
**History of maternal adversity**							
No	46 280	36 490	78.8	28 335	61.2	10 735	23.2
Yes	6275	4845	77.2	4520	72.0	2460	39.2

^a^ *Numbers are rounded to the nearest 5 to align with NHS England guidance for statistical disclosure and therefore may not sum to totals.*

Younger mothers (<24 years) were slightly less likely to receive all three mandated contacts than those aged 30+ (Table 1). However, younger mothers received more additional contacts: nearly half of mothers aged < 20 received three or more additional contacts in their child’s first year. First-time mothers were more likely to receive both mandated and additional contacts than mothers who had previously given birth.

**Fig. 2:**
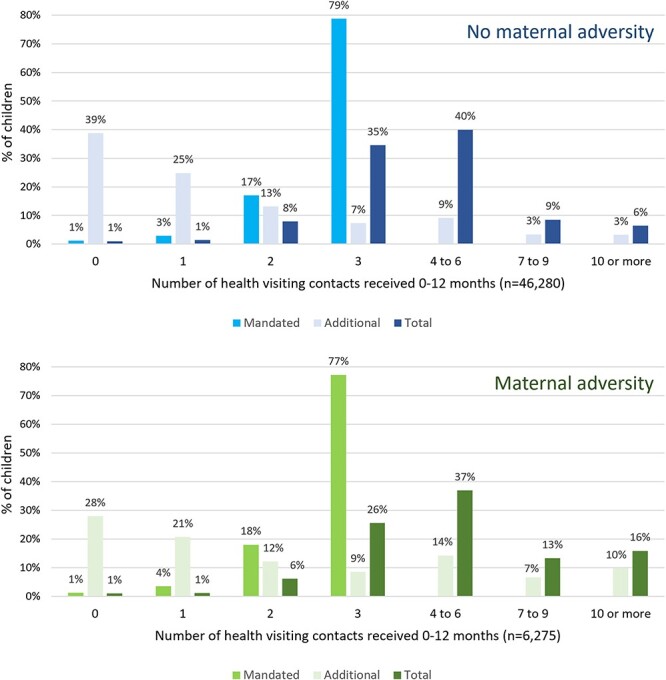
Percentage of children receiving health visiting contacts between birth and age 1 in 10 English local authorities between 2016/17 and 2019/20, by maternal experience of adversity in the 3 years prior to delivery

Of the 52 555 children in our sample, 6275 (11.9%) were born to mothers with a history of adversity-related hospital admissions in the 3 years prior to delivery (Table 1). Children whose mothers had a history of adversity were more likely to receive at least one additional contact (72.0% versus 61.2% in those without maternal adversity) and substantially more likely to receive three or more additional contacts (39.2% compared with 23.2% in those without maternal adversity). As shown in Fig. 2, 16% of children exposed to maternal adversity had contact with the health visiting team at least 10 times in their first year.

### Which characteristics were associated with health visiting support?

In our adjusted regression models, deprivation, child ethnicity, maternal age and whether the mother had given birth before were associated with receiving all three mandated contacts, receiving at least one additional contact, and receiving at least three additional contacts. Maternal adversity was associated with the likelihood of receiving additional contacts (Table 2).

**Table 2:** Relative risks of children receiving different health visiting contacts between birth and age 1 in 10 English local authorities between 2016/17 and 2019/20, according to family characteristics

	Received all 3 mandated contacts		Received ≥ 1 additional contact		Received ≥ 3 additional contacts	
	aRR (95% CI)^a^	p-value	aRR (95% CI)^a^	p-value	aRR (95% CI)^a^	p-value
**Index of Multiple Deprivation decile**						
Most deprived	0.99 (0.96–1.01)	<0.0001	1.06 (1.00–1.13)	<0.0001	1.15 (0.97–1.37)	<0.0001
2	1.00 (0.99–1.01)		1.13 (1.03–1.23)		1.26 (0.93–1.70)	
3	0.99 (0.98–1.00)		1.11 (1.04–1.19)		1.22 (0.97–1.52)	
4	1.02 (1.01–1.03)		1.06 (1.03–1.08)		1.20 (1.03–1.41)	
5	0.99 (0.95–1.03)		1.07 (1.05–1.10)		1.19 (0.95–1.48)	
6	0.99 (0.97–1.02)		1.04 (1.01–1.07)		1.09 (1.00–1.19)	
7	1.00 (0.98–1.02)		1.05 (1.02–1.08)		1.12 (1.00–1.27)	
8	1.00 (0.98–1.02)		1.05 (1.02–1.08)		1.11 (1.07–1.16)	
9	1.01 (1.00–1.02)		1.02 (1.00–1.04)		1.05 (0.97–1.15)	
Least deprived	ref		ref		ref	
**Child ethnicity**						
White	ref	<0.0001	ref	<0.0001	ref	<0.0001
Mixed	0.99 (0.98–1.01)		1.14 (1.07–1.23)		1.17 (0.99–1.39)	
Asian	0.98 (0.95–1.00)		0.95 (0.92–0.98)		0.76 (0.68–0.85)	
Black	0.94 (0.92–0.96)		0.95 (0.90–1.01)		0.95 (0.86–1.05)	
Other	0.91 (0.87–0.94)		0.93 (0.85–1.02)		0.84 (0.73–0.98)	
**Maternal age (years)**						
<20	0.90 (0.83–0.97)	<0.0001	1.14 (0.96–1.35)	<0.0001	1.55 (0.94–2.56)	<0.0001
20–24	0.94 (0.93–0.95)		1.02 (0.95–1.11)		1.09 (0.92–1.29)	
25–29	0.98 (0.97–0.99)		1.00 (0.98–1.03)		0.99 (0.92–1.06)	
30–34	ref		ref		ref	
35–39	1.00 (0.99–1.01)		1.00 (0.99–1.02)		1.01 (0.97–1.04)	
40+	0.99 (0.96–1.02)		1.10 (1.02–1.17)		1.23 (1.07–1.41)	
**First-time mother**						
Yes	1.05 (1.04–1.07)	<0.0001	1.09 (1.07–1.11)	<0.0001	1.24 (1.15–1.33)	<0.0001
**Maternal adversity**						
Yes	0.99 (0.97–1.01)	0.22	1.15 (1.07–1.23)	<0.0001	1.55 (1.26–1.92)	<0.0001

^a^ *Estimates are adjusted for all other variables in the table.*

After adjusting for other family characteristics, living in a more deprived neighborhood was associated with a moderate increase (up to 12%) in the likelihood of receiving additional health visiting contacts, compared to living in the least deprived decile. Children recorded as being from a Black ethnic group were less likely than White children to receive all three mandated contacts; Asian children were less likely to receive additional contacts.

First-time mothers were estimated to be 25% more likely to receive three or more additional contacts than women who had given birth before (adjusted relative risk [aRR] = 1.24, 95% confidence interval [CI] 1.15–1.33). After controlling for other factors, mothers aged 14–19 were 10% less likely to receive all three mandated contacts than mothers aged 30–34 (aRR = 0.90, 95% CI 0.83–0.97) while mothers aged 40 and over were 23% more likely to receive at least three additional contacts (aRR = 1.23, 95% CI 1.07–1.41).

Children whose mothers had a history of adversity were 15% more likely to receive at least one additional contact (aRR = 1.15, 95% CI 1.07–1.23) and 55% more likely to receive at least three additional contacts (aRR = 1.55, 95% CI 1.26–1.92), compared to children not exposed to maternal adversity.

## Discussion

### Main findings of this study

Our study of more than 50 000 children across 10 LAs in UK represents the first analysis of the total amount of health visiting support that infants receive in the first year of life. We found high and consistent delivery of mandated contacts: almost all children (99%) had at least one mandated contact and almost 80% had all three mandated contacts in their first 12 months, with little variation by family characteristics. Additional support was widespread rather than concentrated among a small proportion of families: 63% of children received at least one additional contact and 25% of children had three or more additional contacts before their first birthday.

Although most children in our sample had repeated contact with health visiting teams, the average cumulative ‘dose’ of contact time was low (median 2 h 35 min over 12 months). However, we found a small group of children (4%) who received intensive health visiting support (≥10 additional contacts with median cumulative duration of 11 h contact time). This dose is similar to that found in sustained nurse home visiting programs for targeted populations.^17^^,^^18^

Our findings reflect the principle of proportionate universalism that underpins the health visiting service in UK: families in more vulnerable circumstances received more contacts in addition to the mandated service.^19^ Of the family characteristics considered in this study, the likelihood of receiving additional health visiting support was greatest among children whose mothers had a record of hospital admissions relating to mental health, substance misuse, domestic violence or self-harm in the three years prior to delivery.

### What is already known on this topic

Previous research has indicated that a considerable amount of health visiting takes place outside of the mandated service. A study of 57 LAs in CSDS found that 80% of LAs delivered more additional than mandated contacts between 2018 and 2020.^8^ The recent ‘Children of the 2020s Birth Cohort Study’ found that 27% of 8733 primary caregivers reported ≥4 contacts (of any type) with a member of the health visiting team in the first 9.5 months of their child’s life.^20^

At the same time, reports from the front line of health visiting suggest that the service is struggling to provide support to families beyond the universal service, except to those with the most complex needs. In the 2023 Institute of Health Visiting (iHV) survey, 45% of 1186 health visitors reported that mandated contacts and child protection were routinely prioritized over other preventive work.^3^ The 2018 iHV survey noted that ‘Health visitors are now “walking a very tight rope” between being strongly driven to meet KPIs (key performance indicators) […] on the one hand and child protection social work-by-proxy on the other’.^21^

### What this study adds

Our study demonstrates that health visiting in UK is not confined to the mandated service: in 10 LAs between 2016 and 2020, 63% of families were able to access additional health visiting support and up to 50% of children in the most vulnerable circumstances (living in deprived areas, with young mothers and/or exposed to maternal adversity) had three or more contacts with the health visiting team in addition to their mandated reviews.

However, this means that many children who may have been vulnerable—for example, 28% of children whose mothers had a history of hospital admissions relating to mental health, substance misuse, domestic violence or self-harm—did not receive any additional health visiting support in the first year of life. This may have been because those families did not experience any major difficulties during that time; however, it may have been because their local health visiting team lacked the resources to respond to the needs of all families in the area.

### Limitations of this study

A major limitation is that we could only include 10 local areas with sufficiently complete longitudinal data in our study sample. The majority of children in the sample were from one LA, which limits generalizability of the cohort to UK more widely, and we show differences in deprivation and ethnic diversity in these 10 LAs compared to UK. These LAs were selected on the basis of data completeness, which may reflect more operational capacity generally and therefore greater service delivery than in other areas. A priority for future health visiting research is to enable LAs to submit more complete data to CSDS.

Our data reflected the situation before the COVID-19 pandemic; with increasing funding and workforce challenges, it is possible that the reach of health visiting teams is now lower than we found in 2016–20.^14^ The proportion of children who received little or no health visiting support in the first 12 months may have been higher during the pandemic (where lockdowns prevented routine health visiting services) than in our sample.

Our sample excluded children who were born outside NHS hospitals; children who moved LA in the first 12 months; and children who were not registered in CSDS at any point between 2016 and 2020. These may further restrict the generalizability of our findings.

Finally, we had no information on the reasons why families receive additional health visiting contacts; in particular, we do not know whether additional contacts were provided in response to a family request or initiated by the health visiting team because of perceived need. Collection of such data would help to illuminate the balance of mandated, responsive and preventive health visiting work in different areas.

Despite these limitations, our study indicates that need for health visiting support over and above the mandated service was high from 2016 to 2020, with intensive support concentrated among vulnerable children, particularly those whose mothers had a history of adversity. It is likely that need has increased in recent years with declining living standards, greater prevalence of parental mental health problems and increasingly complex and entrenched problems among families with young children.^22^ Policymakers and commissioners should consider how health visiting services can be expanded or targeted more effectively to ensure all families receive the support they need in the early weeks and months of a child’s life.

## Supplementary Material

Health_visiting_support_by_family_characteristics_suppl_230824_fdae259

## Data Availability

The data underlying this article cannot be shared publicly due to the terms of the data sharing agreement with NHS England. Data can be obtained by submitting a data request through the NHS England Data Access Request Service.

## Notes

**C Bunting,** Research Assistant

**A Clery,** Research Fellow

**L McGrath-Lone,** Lecturer

**M Liu,** Research Fellow

**S Kendall,** Professor

**H Bedford,** Professor

**F Cavallaro,** Principal Data Analyst

**EC Saloniki,** Senior Research Fellow

**K Harron,** Professor

**J Woodman,** Associate Professor
