# Supplementary material for: How does health visiting in the first year of life vary by family characteristics? A longitudinal analysis of administrative data
Source: J Public Health (Oxf). 2024 Sep 16;47(1):82–9. doi: 10.1093/pubmed/fdae259 (PMC11879038; doi:10.1093/pubmed/fdae259)
Supplement: Health_visiting_support_by_family_characteristics_suppl_230824_fdae259 [file health_visiting_support_by_family_characteristics_suppl_230824_fdae259.docx]

How does health visiting in the first year of life vary by family characteristics? A longitudinal analysis of administrative data

# Supplementary appendices

**Supplementary Figure 1: Cohort creation flow diagram**

**
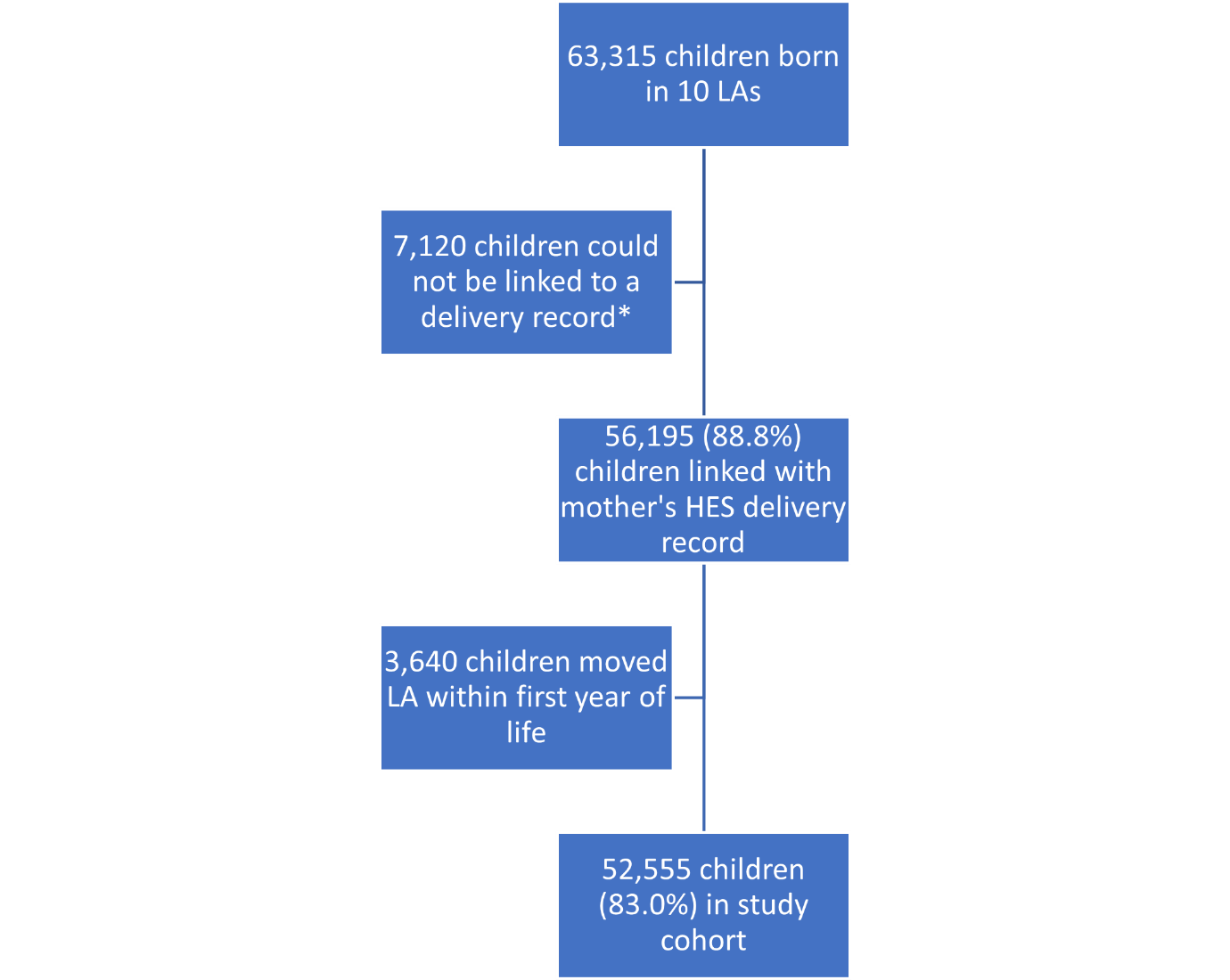
**

** Children may not have linked to a delivery record if they were born outside of the UK or not in a NHS hospital; or as a result of linkage error, for example due to missing data on matching variables.*

**Supplementary Table 1: Breakdown of study sample by local authority**

| **Local authority** | **Number of children** | **% of sample** | **Birth window** |
| --- | --- | --- | --- |
| 1 | 1,080 | 2.1% | Oct 17 - Dec 17 |
| 2 | 1,690 | 3.2% | Apr 18 - Jun 18 |
| 3 | 35,845 | 68.2% | Apr 16 - Mar 19 |
| 4 | 1,235 | 2.3% | Apr 17 - Jun 17 |
| 5 | 1,405 | 2.7% | Apr 16 - Mar 19 |
| 6 | 1,480 | 2.8% | Oct 18 - Mar 19 |
| 7 | 825 | 1.6% | Apr 17 - Jun 17 |
| 8 | 1,905 | 3.6% | Oct 18 - Mar 19 |
| 9 | 6,355 | 12.1% | Jul 17 - Mar 19 |
| 10 | 735 | 1.4% | Mar 18 – Jun 19 |

**Supplementary Table 2: Code lists for maternal adversity**

Maternal adversity includes women who had experienced at least one episode relating to mental health *or* at least one unplanned episode relating to substance misuse, violence or self-harm in the three years prior to delivery.

***Mental Health***

The following ICD-10 diagnosis codes, in any diagnostic position, were used to identify mental health diagnoses in any type of admission:

| **Category** | **Sub-category** | **ICD-10 codes** | **ICD-10 Description** |
| --- | --- | --- | --- |
| Internalising | Mood Disorders | F320 | Mild depressive episode |
|  |  | F321 | Moderate depressive episode |
|  |  | F322 | Severe depression without psychotic symptoms |
|  |  | F328 | Other depressive episodes |
|  |  | F329 | Depressive episode, unspecified |
|  |  | F330 | Recurrent depressive disorder, current episode mild |
|  |  | F331 | Recurrent depressive disorder, current episode moderate |
|  |  | F332 | Recurrent depressive disorder, current episode severe without psychotic symptoms |
|  |  | F333 | Recurrent depressive disorder, current episode severe with psychotic symptoms |
|  |  | F338 | Other recurrent depressive disorders |
|  |  | F339 | Recurrent depressive disorder, unspecified |
|  |  | F340 | Cyclothymia |
|  |  | F341 | Dysthymia |
|  |  | F348 | Other persistent mood (affective) disorders |
|  |  | F349 | Persistent mood (affective) disorder, unspecified |
|  |  | F380 | Other single mood (affective) disorders |
|  |  | F381 | Other recurrent mood (affective) disorders |
|  |  | F388 | Other specified mood (affective) disorders |
|  |  | F39 | Unspecified mood (affective) disorder |
|  | Anxiety or fear-related, Obsessive-compulsive, & Dissociative disorders | F40 | Phobic anxiety disorders |
|  |  | F41 | Other anxiety disorders |
|  |  | F42 | Obsessive-compulsive disorder |
|  |  | F44 | Dissociative (conversation) disorders |
|  |  | F45 | Somatoform disorders |
|  |  | F48 | Other neurotic disorders |
|  |  | F54 | Psychological and behavioural factors associated with disorders or diseases classified elsewhere |
|  |  | F94.0 | Elective mutism |
|  | Disorders specifically associated with stress | F43 | Reaction to severe stress, and adjustment disorders |
|  |  | F62 | Enduring personality changes, not attributable to brain damage and disease |
|  |  | F94.1 | Reactive attachment disorder of childhood |
|  |  | F94.2 | Disinhibited attachment disorder of childhood |
|  | Eating disorders | F50 | Eating disorders |
|  | Sleep disorders | F51 | Non-organic sleep disorders |
|  | Abuse of non-dependence-producing substances | F55 | Abuse of non-dependence-producing substances |
|  | Emotional disorders with onset usually occurring in childhood and adolescence | F93 | Emotional disorders with onset specific to childhood |
|  |  | F94 | Disorders of social functioning with onset specific to childhood and adolescence |
|  |  | F98 | Other behavioural and emotional disorders with onset usually occurring in childhood and adolescence |
| Externalising | Impulse control, Disruptive behaviour or dissocial disorders | F63 | Habit and impulse disorders |
|  |  | F90 | Disturbance of activity and attention |
|  |  | F91 | Conduct disorders |
|  |  | F92 | Mixed disorders of conduct and emotions |
| Thought Problems & Personality Disorders | Mood Disorders with mania or psychotic symptoms | F30 | Manic episode |
|  |  | F31 | Bipolar affective disorder |
|  |  | F323 | Severe depression with psychotic symptoms |
|  | Schizophrenia or other primary psychotic disorders | F20 | Schizophrenia |
|  |  | F21 | Schizotypal disorder |
|  |  | F22 | Persistent delusional disorders |
|  |  | F23 | Acute and transient psychotic disorders |
|  |  | F24 | Induced delusional disorder |
|  |  | F25 | Schizoaffective disorders |
|  |  | F28 | Other nonorganic psychotic disorders |
|  |  | F29 | Unspecified nonorganic psychosis |
|  | Disorders of adult personality and behaviour | F60 | Specific Personality Disorders |
|  |  | F61 | Mixed and other personality disorders |
|  |  | F68 | Other disorders of adult personality and behaviour |
|  |  | F69 | Unspecified disorder of adult personality and behaviour |
| Mental health puerperium | Mental & behavioural disorders associated with the puerperium | F53 | Mental & behavioural disorders associated with the puerperium |

***Substance misuse, violence and self-harm***

Admissions relating to substance misuse, violence and self-harm were flagged where an episode contained one of the following ICD-10 codes, in any diagnostic position, and the patient was admitted as an emergency (Admimeth= 21-25, 28, 2A or 2D).

| ***Category*** | ***ICD-10 Codes*** | ***ICD-10 Description*** |
| --- | --- | --- |
| Drug and/or alcohol misuse | F10 | Mental and behavioural disorders due to use of alcohol |
|  | F11-16, F18-19 | Mental and behavioural disorders due to psychoactive substance use |
|  | R78.0 | Finding of alcohol in blood |
|  | R78.1- R78.5 | Findings of drugs and other substances, not normally found in blood |
|  | T36-T50 | Poisoning by drugs, medicaments and biological substances |
|  | X45 | Accidental poisoning by and exposure to alcohol |
|  | X40-X44 X46-X49 | Accidental poisoning by and exposure to noxious substances |
|  | X69 | Intentional self-poisoning by and exposure to other and unspecified chemicals and noxious substances |
|  | Y10-Y14, Y16-Y19 | Poisoning, undetermined intent |
|  | Y15 | Poisoning by and exposure to alcohol, undetermined intent |
|  | Y90 | Evidence of alcohol involvement determined by blood alcohol level |
|  | Y91 | Evidence of alcohol involvement determined by level of intoxication |
|  | Z50.2 | Alcohol rehabilitation |
|  | Z50.3 | Drug rehabilitation |
|  | Z71.4 | Alcohol abuse counselling and surveillance |
|  | Z71.5 | Drug abuse counselling and surveillance |
|  | Z72.1 | Problems related to lifestyle, alcohol use |
|  | Z72.2 | Problems related to lifestyle, drug use |
|  | G40.5 | Special epileptic syndromes |
|  | Z04.0 | Examination for Blood-alcohol and blood-drug test |
|  | O35.4 | Maternal care for (suspected) damage to foetus from alcohol |
|  | E24.4 | Alcohol-induced pseudo-Cushing syndrome |
|  | G31.2 | Degeneration of nervous system due to alcohol |
|  | G62.1 | Alcoholic polyneuropathy |
|  | G72.1 | Alcoholic polyneuropathy |
|  | I42.6 | Alcoholic cardiomyopathy |
|  | K29.2 | Alcoholic gastritis |
|  | K70 | Alcoholic liver disease |
|  | K85.2 | Alcohol-induced acute pancreatitis |
|  | K86.0 | Alcohol-induced chronic pancreatitis |
|  | T51 | Toxic effect of alcohol |
| Violence (including domestic violence) | T74 | Maltreatment syndrome |
|  | T73 | Effects of other deprivation (extreme neglect) |
|  | Y06, Y07 | Perpetrator of neglect and other maltreatment syndromes |
|  | Y04, Y05 | Assault by bodily force and sexual assault |
|  | X85- Y03, Y08, Y09 | Other type of assault |
|  | Y20-Y34 | Events of undetermined intent |
|  | Z04.5 | Examination and observation following other inflicted injury |
|  | Z04.8 | Examination and observation for other reasons: request for expert evidence |
|  | Z63.0 | Problems in relationship with spouse or partner |
| Self Harm | X60-68 | Intentional self-poisoning |
|  | X70-X84 | Intentional self-harm |
|  | Y87.0 | Sequelae of intentional self-harm |
|  | Z91.5 | Personal history of self-harm |

**Supplementary Table 3: Characteristics of the 10 local authorities included in the longitudinal cohort and all other local authorities in England**

|  | **All local authorities in England**  **(N=149)** | | **Local authorities in longitudinal cohort**  **(N=10)** | | **p-value^a^** |
| --- | --- | --- | --- | --- | --- |
|  | **n** | **%** | **n** | **%** |  |
| **Region** | | | | | |
| East Midlands | 9 | 6.0 | 1 | 10.0 | 0.35 |
| East of England | 11 | 7.4 | 1 | 10.0 |  |
| London | 32 | 21.5 | 0 | 0.0 |  |
| North East | 12 | 8.1 | 0 | 0.0 |  |
| North West | 23 | 15.4 | 2 | 20.0 |  |
| South East | 19 | 12.8 | 1 | 10.0 |  |
| South West | 14 | 9.4 | 2 | 20.0 |  |
| West Midlands | 14 | 9.4 | 1 | 10.0 |  |
| Yorkshire and The Humber | 15 | 10.1 | 2 | 20.0 |  |
| **Rural-Urban classification^b^** | | | | | |
| Predominantly Rural | 20 | 13.4 | 2 | 20 | 0.12 |
| Predominantly Urban | 108 | 72.5 | 5 | 50 |  |
| Urban with significant rural | 21 | 14.1 | 3 | 30 |  |
| **Index of multiple deprivation^c^** | | | | | |
| Lowest quintile | 30 | 20.1 | 1 | 10 | 0.71 |
| 2^nd^ quintile | 30 | 20.1 | 1 | 10 |  |
| 3^rd^ quintile | 30 | 20.1 | 3 | 30 |  |
| 4^th^ quintile | 30 | 20.1 | 2 | 20 |  |
| Highest quintile | 29 | 19.5 | 3 | 30 |  |
| **Income deprivation affecting children index^c^** | | | | | |
| Lowest quintile | 30 | 20.1 | 1 | 10 | 0.71 |
| 2^nd^ quintile | 30 | 20.1 | 1 | 10 |  |
| 3^rd^ quintile | 30 | 20.1 | 2 | 20 |  |
| 4^th^ quintile | 30 | 20.1 | 3 | 30 |  |
| Highest quintile | 29 | 19.5 | 3 | 30 |  |
| **Ethnic distribution^d^** | | | | | |
| Asian (median %; IQR) | 7.2; 2.6-13.1 | | 3.7; 2.4-5.3 | | 0.04 |
| Black (median %; IQR) | 2.3; 0.8-7.4 | | 1.1; 0.7-1.3 | | 0.08 |
| Mixed (median %; IQR) | 2.7; 1.6-4.3 | | 1.8; 1.5-2.4 | | 0.07 |
| Other (median %; IQR) | 1.5; 0.8-3.3 | | 0.9; 0.7-1.4 | | 0.10 |
| White (median %; IQR) | 85.3; 67.8-94.1 | | 93.0; 88.6-94.2 | | 0.05 |

*^a^Testing the difference between the local authorities included vs. not included in the longitudinal cohort. P-values for categorical values calculated using Fisher’s exact test. P-values for continuous variables calculated using Mann-Whitney U-test.*

*^b^2011 3-fold classification;* [*https://www.gov.uk/government/statistics/2011-rural-urban-classification-lookup-tables-for-all-geographies*](https://www.gov.uk/government/statistics/2011-rural-urban-classification-lookup-tables-for-all-geographies)

*^c^2019 quintiles summarised from individual score ranks;* [*https://www.gov.uk/government/statistics/english-indices-of-deprivation-2019*](https://www.gov.uk/government/statistics/english-indices-of-deprivation-2019)

*^d^2021 Census percentage of the population by ethnic group;* [*https://www.ons.gov.uk/peoplepopulationandcommunity/culturalidentity/ethnicity/bulletins/ethnicgroupenglandandwales/census2021*](https://www.ons.gov.uk/peoplepopulationandcommunity/culturalidentity/ethnicity/bulletins/ethnicgroupenglandandwales/census2021)
